# An Electrocardiographic Characterization of Left Bundle Branch Area Pacing-Induced Right Ventricular Activation Delay: A Comparison With Native Right Bundle Branch Block

**DOI:** 10.3389/fcvm.2022.885201

**Published:** 2022-06-09

**Authors:** Emine Ozpak, Anthony Demolder, Sevda Kizilkilic, Simon Calle, Frank Timmermans, Jan De Pooter

**Affiliations:** Heart Center, Ghent University Hospital, Ghent, Belgium

**Keywords:** left bundle branch area pacing, conduction system pacing, cardiac pacing, right ventricular activation, ventricular activation time

## Abstract

**Background:**

Left bundle branch area pacing (LBBAP) induces delayed RV activation and is thought to be harmless, since the electrocardiographic signature is reminiscent to native RBBB. However, to what extent the delayed RV activation during LBBAP truly resembles that of native RBBB remains unexplored.

**Methods:**

This study included patients with incomplete RBBB (iRBBB), complete RBBB (cRBBB) and patients who underwent LBBAP. Global and right ventricular activation times were estimated by QRS duration and R wave peak time in lead V1 (V1RWPT) respectively. Delayed RV activation was further characterized by duration, amplitude and area of the terminal R wave in V1.

**Results:**

In patients with LBBAP (*n* = 86), QRS duration [120 ms (116, 132)] was longer compared to iRBBB patients (*n* = 422): 104 ms (98, 110), *p* < 0.001, but shorter compared to cRBBB (*n* = 223): 138 ms (130, 152), *p* < 0.001. V1RWPT during LBBAP [84 ms (72, 92)] was longer compared to iRBBB [74 ms (68, 80), *p* < 0.001], but shorter than cRBBB [96 ms (86, 108), *p* < 0.001]. LBBAP resulted in V1 R′ durations [42 ms (28, 55)] comparable to iRBBB [42 ms (35, 49), *p* = 0.49] but shorter than in cRBBB [81 ms (68, 91), *p* < 0.001]. During LBBAP, the amplitude and area of the V1 R′ wave were more comparable with iRBBB than cRBBB. V1RWPT during LBBAP was determined by baseline conduction disease, but not by LBBAP capture type.

**Conclusion:**

LBBAP-induced delayed RV activation electrocardiographically most closely mirrors the delayed RV activation as seen with incomplete rather than complete RBBB.

## Introduction

Right ventricular (RV) apex pacing has been considered the standard pacing approach since its first attempt in 1958 (1). Although this pacing strategy meets its primary objective (pacing the heart), it can induce a dyssynchronous ventricular activation which can lead to pacing-induced cardiomyopathy, adverse cardiac remodeling and increased mortality (2–8). Conduction system pacing (CSP) recently emerged as an alternative pacing approach to achieve physiological pacing and it may avoid the detrimental effects of RV pacing. Among the CSP modalities, His bundle pacing (HBP) is considered the most physiological since it optimally mimics the normal cardiac conduction, but it is limited by high capture thresholds, low sensing amplitudes and low implant success in patients with infranodal conduction disease (9, 10). On the other hand, left bundle branch area pacing (LBBAP) is a novel approach to achieve physiological pacing and has more favorable pacing characteristics (i.e., lower pacing thresholds and higher sensing amplitudes) compared to HBP (11–15). LBBAP aims to capture the left bundle branch (LBB) and results in a fast and homogenous activation of the left ventricle (LV) comparable to HBP (16). In contrast, activation of the RV is delayed, which is not the case in HBP. This delayed RV activation is electrocardiographically characterized by a right bundle branch block (RBBB) pattern on the electrocardiogram (ECG), and is considered one of the hallmarks of successful LBBAP (17). In patients without structural heart disease, delayed RV activation due to native RBBB is generally considered benign as it does not result in adverse outcome (18–23), and therefore it can be postulated that LBBAP-induced delayed RV activation is probably benign. However, to what extent LBBAP-induced delayed RV activation truly resembles native RBBB activation in healthy individuals is currently unknown. This study aims to compare the electrocardiographic characteristics of delayed RV activation in patients with native RBBB vs. patients with LBBAP-induced RBBB-like ECG pattern.

## Methods

### Study Design

The study enrolled consecutive adult in- and outpatients diagnosed with either incomplete RBBB (iRBBB) or complete RBBB (cRBBB) on standard twelve-lead ECG between January 2015 and September 2018. LBBAP patients implanted between March 2020 and October 2021 were included in the LBBAP group.

All patients were recruited at the Ghent University Hospital. The study was approved by the Ethics Committee of the Ghent University Hospital.

### Selection of iRBBB and cRBBB Patients

Contemporary definitions of iRBBB and cRBBB were used to select RBBB patients. QRS duration cut-offs used for iRBBB and cRBBB were 110–119 and ≥120 ms respectively (22). Patients with iRBBB and cRBBB were identified by the Marquette 12SL algorithm (GE Healthcare, Chicago, IL, United States) in the Muse ECG database (GE Healthcare).

### LBBAP Implant and Definition of Capture Type

LBBAP implant was performed as previously described and both lumen-less and conventional stylet-driven pacing leads were used (17, 24). Successful LBBAP was defined as either conduction system capture (left bundle branch pacing, LBBP) or myocardial capture (left ventricular septal pacing, LVSP). Following criteria were used to define the type of capture (15, 17, 25, 26): (1) appearance of a Qr, qR, rSr pattern in lead V1, (2) observed transition in pacing responses (non-selective, selective LBBP or myocardial capture) with changes in unipolar pacing output, (3) stimulus to R wave peak time in lead V6 <75 ms in patients with baseline narrow QRS or RBBB or ≤ 80 ms in patients with left bundle branch block (LBBB) or intraventricular conduction delay (IVCD) (26). Patients that fulfilled the first criterium and at least one additional criterium were considered LBBP; if only the first criterium was met, the pacing response was defined as LVSP (17).

### Electrocardiographic Analysis

ECG's were recorded at a paper speed of 25 mm/s and a calibration of 10 mm/mV with MAC 5500 ECG recording devices (GE Healthcare). To avoid any fusion with intrinsic rhythm during LBBAP, paced QRS morphologies were obtained during VVI pacing with a lower rate 20–30 beats higher then intrinsic heart rate. Global ventricular activation was measured as global QRS duration, in which the QRS was measured from its onset to the latest QRS offset in any lead (22, 27). The right ventricular activation time (RVAT) was estimated by the R wave peak time in lead V1 (V1RWPT), measured from QRS onset to the peak of the R wave in lead V1 (i.e., the R wave in case of qR pattern and the r′ wave in rSr′ pattern). Left ventricular activation time (LVAT) was calculated from QRS onset to the R wave peak in lead V6 (V6RWPT) (26, 28). The interval between the R wave peak time in V6 and V1 was defined as the V6–V1 interpeak interval (V6V1 IPI) and used as an estimation of interventricular electrical dyssynchrony (29).

All electrocardiographic measurements were performed with digital calipers and adapted sweep speeds of 50 mm/s on the digitally stored ECG's. The delayed RV activation was further characterized by measuring the duration, amplitude and area of the delayed R wave in V1 using automated measurements provided by the 12SL algorithm (GE Healthcare) (30).

### Statistical Analysis

Categorical variables are expressed as absolute number (percentage). Continuous variables are expressed as mean ± standard deviation in case of Gaussian distribution or median [1st; 3rd quartile] if data follow a non-Gaussian distribution. Normality was tested using the Shapiro–Wilk test. To compare means and medians of continuous variables among groups the one-way ANOVA and Kruskall Wallis test was used. The Wilcoxon signed rank test was used for paired comparison of non-Gaussian distributed continuous variables. Multivariate analysis was performed to assess determinants of delayed RV activation using multiple regression analysis. Statistical significance was set at a two-tailed probability level of < 0.05. All statistical analyses were performed using SPSS software (version 28.0, IBM, Armonk, NY, United States).

## Results

### Patient Characteristics

Overall, the study included 731 patients with delayed RV activation: 422 patients with iRBBB, 223 patients with cRBBB and 86 patients with LBBAP. Baseline patient characteristics are summarized in Table 1.

**Table 1:** Baseline patient characteristics.

	**iRBBB (*n* = 422)**	**cRBBB (*n* = 223)**	**LBBAP (*n* = 86)**	***p*** **-value**
**Baseline patient characteristics**				
Age, years	51 ± 18	57 ± 21	69 ± 16	*p* <0.001
Female gender, *n* (%)	282 (67)	164 (74)	61 (71)	*p* = 0.20
Weight, kg	75 ± 15	76 ± 19	80 ± 19	*p* = 0.05
Length, cm	174 ± 10	170 ± 10	169 ± 13	*p* <0.001
**Medical history**				
Ischemic heart disease, *n* (%)	72 (17%)	46 (21%)	24 (28%)	*p* <0.001
Acute coronary syndrome, *n* (%)	12 (3%)	46 (21%)	10 (12%)	*p* <0.001
Heart failure, *n* (%)	24 (6%)	46 (21%)	7 (8%)	*p* <0.001
Atrial fibrillation, *n* (%)	4 (2%)	4 (1%)	32 (37%)	*p* <0.001
**Echocardiographic characteristics**				
Left atrial diameter, mm	36 ± 7	40 ± 9	40 ± 9	*p* <0.001
Left ventricular end diastolic diameter, mm	46 ± 6	48 ± 7	49 ± 9	*p* = 0.007
**Electrocardiographic characteristics**				
QRS duration, ms	104 (98, 110)	138 (130, 152)	112 ms (94, 147)	*p* <0.001

*Continuous variables are expressed as mean ± standard deviation. Categorical variables are expressed as number of patients (percentage)*.

*iRBBB, incomplete right bundle branch block; cRBBB, complete right bundle branch block. LBBAP, left bundle branch area pacing*.

In patients who underwent LBBAP, pacing indication was atrioventricular block in 62%, brady-tachy syndrome in 17%, sinus node disease in 16% and heart failure in 5%. In patients undergoing LBBAP, baseline QRS measured 112 ms (94, 147), with 55% having narrow QRS, 14.5% left bundle branch block (LBBB), 14.5% RBBB and 17% non-specified intraventricular conduction delay (NIVCD). LBBAP pacing response was labeled as LBBP (non-selective and selective) in 62 (72%) patients, whereas LVSP was achieved in 24 (28%) patients.

### Ventricular Activation Times During LBBAP in Comparison to iRBBB and cRBBB Patients

**Figure 1:**
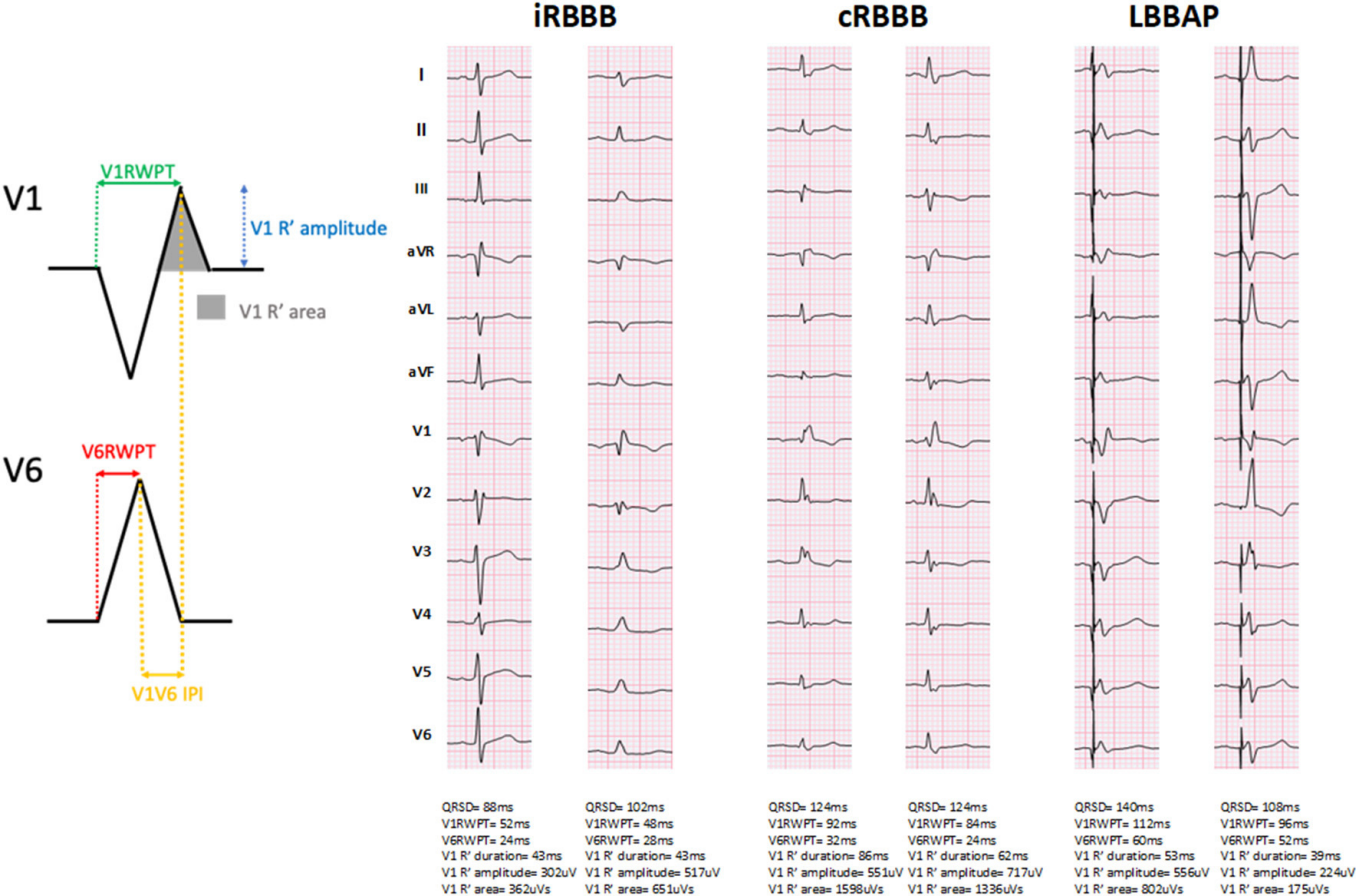
Representative examples of ventricular activation times measured during incomplete right bundle branch block (iRBBB), complete right bundle branch block (cRBBB) and left bundle branch area pacing (LBBAP). QRSD: QRS duration. V1RWPT, V1 R-wave peak time; V6RWPT, V6 R-wave peak time; V6V1 IPI, V6-V1 interpeak interval.

**Figure 2:**
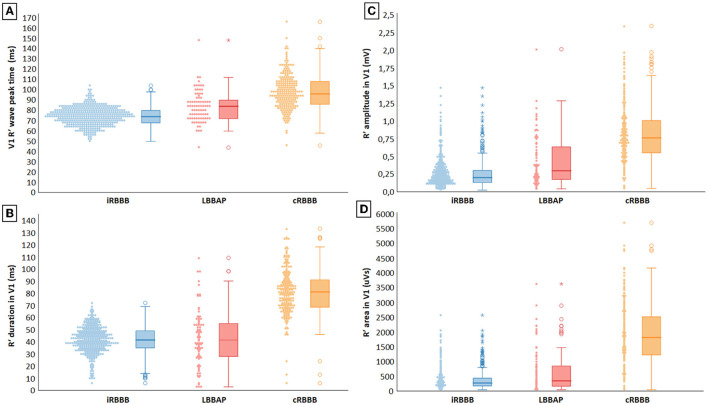
**(A–D)** Electrocardiographic characterization of delayed RV activation during incomplete right bundle branch block (iRBBB), complete right bundle branch block (cRBBB) and left bundle branch area pacing (LBBAP).

Representative examples of ventricular activation time measurements with iRBBB, cRBBB and LBBAP are shown in Figure 1. Paced QRS duration during LBBAP was 120 ms (116, 132), whereas QRS duration of iRBBB and cRBBB patients was 104 ms (98, 110) and 138 ms (130, 152) respectively (*p* < 0.001). V1RWPT during LBBAP was 84 ms (72, 92) and longer compared to iRBBB patients [74 ms (68, 80), *p* < 0.001], but shorter in comparison to cRBBB patients [96 ms (86, 108), *p* < 0.001] (Figure 2A). V6RWPT during LBBAP [44 ms (36, 56)] was only slightly longer than V6RWPT measured during iRBBB [40 ms (36, 44), *p* < 0.001] and cRBBB [38 ms (36, 44), *p* < 0.001]. The V6V1 IPI for LBBAP patients was comparable to iRBBB patients [36 ms (24, 45) and 34 ms (28, 40), respectively; *p* = 0.70]. Compared to cRBBB, the V6V1 IPI was shorter for LBBAP patients [58 ms (48, 68) vs. 36 ms (24, 45), *p* < 0.001].

R′ duration in V1 with LBBAP-induced RBBB was 42 ms (28, 55) and was comparable to V1 R′ duration in iRBBB patients [42 ms (35, 49), *p* = 0.49], but shorter than in cRBBB patients [81 ms (68, 91), *p* < 0.001] (Figure 2B). Mean V1 R′ amplitude during LBBAP measured 297 uV (175, 645), which was also smaller compared to cRBBB patients, but larger than iRBBB patients [respectively 761 uV (551, 1,010) and 195 uV (126, 298), *p* < 0.001] (Figure 2C). R′ area in V1 during LBBAP [316 uVs (134, 831)] was smaller compared to cRBBB [1,782 uVs (1,182, 2,498), *p* < 0.001], but larger than in patients with iRBBB [236 uVs (140, 399), *p* = 0.008] (Figure 2D).

### Ventricular Activation Times During LBBAP According to Baseline Conduction Disease

With LBBAP, QRS duration shortened from 153 ms (142, 160) to 116 ms (104, 136) (*p* < 0.001) in patients with LBBB, from 147 ms (137, 158) to 136 ms (122, 136) (*p* = 0.009) in RBBB patients and from 135 ms (128, 153) to 128 ms (118, 133) (*p* = 0.33) in patients with NIVCD. In patients with baseline narrow QRS (<120 ms), QRS duration increased from 94 ms (84, 106) to 120 ms (115, 128) (*p* < 0.001) with LBBAP. Ventricular activation times during LBBAP according to baseline conduction disease are summarized in Table 2. The longest V1RWPT were observed in LBBAP patients with pre-existing RBBB [84 ms (72, 92)] and NIVCD [90 ms (83, 100)], although the differences with narrow QRS [82 ms (72, 89)] and LBBB [72 ms (65, 79)] patients were small (*p* = 0.014). R′ duration in V1 was significantly shorter for patients with narrow QRS [39 ms (20, 52)] undergoing LBBAP, compared to LBBAP patients with underlying RBBB [46 ms (38, 74)], LBBB [48 ms (38, 55)] and NIVCD [49 ms (35, 66)], *p* = 0.04. Of interest, in LBBAP patients with presumed delay of the right bundle branch conduction (such as RBBB and NIVCD patients), the V1RWPT and V1 R′ duration were still shorter compared to cRBBB patients (*p* = 0.03 and *p* < 0.001, respectively).

**Table 2:** Ventricular activation times during native conduction and LBBAP according to baseline conduction disease.

	**V6RWPT (ms)**	**V1RWPT (ms)**	**V1 R^′^duration (ms)**	**V1 R^′^amplitude (uV)**	**V1 R^′^area (uVs)**	**V6V1 IPI**
iRBBB (*n* = 422)	40 (36, 44)	74 (68, 80)	42 (35, 49)	195 (126, 298)	236 (140, 399)	34 (28, 40)
cRBBB (*n* = 223)	38 (36, 44)	96 (86, 108)	81 (68, 91)	761 (551, 1010)	1782 (1182, 2498)	58 (48, 68)
LBBAP mean (*n* = 86)	44 (36, 56)	84 (72, 92)	42 (28, 55)	297 (175, 645)	316 (134, 831)	36 (24, 45)
LBBAP: narrow QRS (*n* = 47)	44 (36, 56)	82 (72, 89)	39 (20, 52)	246 (144, 504)	263 (213, 418)	32 (24, 45)
LBBAP: LBBB (*n* = 12)	40 (32, 44)	72 (65, 79)	48 (38, 55)	449 (217, 782)	509 (94, 428)	32 (24, 44)
LBBAP: RBBB (*n* = 12)	46 (29, 62)	84 (72, 92)	46 (28, 74)	251 (135, 652)	325 (205, 451)	40 (24, 56)
LBBAP: NIVCD (*n* = 15)	46 (40, 62)	90 (83, 100)	49 (35, 66)	530 (240, 871)	798 (241, 448)	38 (32, 58)

*iRBBB, incomplete right bundle branch block; cRBBB, complete right bundle branch block; LBBAP: left bundle branch area pacing; RBBB, right bundle branch block; LBBB, left bundle branch block; NIVCD, non-specified intraventricular conduction delay; V6V1 IPI, V6-V1 interpeak interval*.

### Ventricular Activation Times During LBBAP According to LBBAP Capture Type

V1RWPT values were comparable for patients with LBBP (*n* = 62) and LVSP (*n* = 24): 84 ms (72, 88) vs. 80 ms (72, 91), respectively (*p* = 0.43); and V1RWPT of both LBBP and LVSP patients resembled more V1RWPT of iRBBB patients compared to cRBBB patients.

V6RWPT values were shorter in LBBP patients [42 ms (33, 54)] compared to patients with LVSP capture type [52 ms (45, 62), *p* = 0.01]. Due to the comparable V1RWPT but different V6RWPT, patients with LBBP presented with longer V6V1 IPI compared to LVSP [40 ms (32, 48) vs. 26 ms (24, 37), respectively, *p* = 0.001].

In patients with LBBP, V1 R′ duration, amplitude and area [respectively 42 ms (27, 58), 337 uV (193, 686), 332 uVs (231, 418)] were comparable to LVSP [45 ms (31, 54), *p* = 0.45; 254 uV (168, 523), *p* = 0.76; 285 uVs (129, 494), *p* = 0.45).

### Determinants of Delayed Right Ventricular Activation in iRBBB, cRBBB and LBBAP Patients

Due to differences in baseline characteristics between iRBBB, cRBBB and LBBAP patients (Table 1), determinants of delayed RV activation were analyzed. In univariate analysis ischemic heart disease, history of atrial fibrillation and presence of heart failure were associated with longer V1RWPT and V1 R′ duration among the entire population of iRBBB, cRBBB and LBBAP patients. However, in a multiple regression analysis only the presence of heart failure and patient group (iRBBB, cRBBB and LBBAP) remained significant and independently associated with V1RWPT and V1 R′ duration.

## Discussion

### Main Findings

This study is the first to compare the electrocardiographic pattern of LBBAP-induced delayed RV activation to the delayed RV activation observed in patients with conduction delay of the right bundle branch. Our results show that the delayed RV activation during LBBAP electrocardiographically mirrors more closely to native iRBBB than cRBBB, with activation times in between those of iRBBB and cRBBB patients. With LBBAP, the delayed RV activation seems to be determined by the underlying conduction disease rather than the type of LBBAP capture (i.e., conduction system capture vs. myocardial capture).

### Left Bundle Branch Area Pacing and Left and Right Ventricular Activation Times

LBBAP aims to capture the left bundle branch itself (LBBP) or the left-sided septal myocardium (LVSP) in the direct area of the left bundle branch. Several studies investigated the contraction and activation patterns of the left ventricle (LV) during LBBAP and revealed a fast and homogenous activation of the LV resulting in beneficial hemodynamic effects of LBBAP (12, 15). Although LV activation during LBBP seems to occur earlier compared to LVSP, differences are small and may not be clinically relevant. This could be explained by the deep left-sided septal position of the pacing lead that quickly activates the adjacent left-sided conduction fibers. Indeed, at the left side of the septum, the LBB is a widely arborized structure and a pacing lead with a deep septal position is more likely to be embedded in close proximity to the conduction system, resulting in a homogenous LV activation (12, 15). With LBBAP, the fast LV activation is estimated by the so-called LVAT or either R wave peak time in lead V6 of the ECG and is often used to define successful LBBAP (26, 28). Both measurements are used interchangeably and assess the interval between the pacing stimulus or QRS onset and the R wave peak time in lead V4, V5 or V6. The shorter these intervals, the faster and probably more homogenous the LV is thought to be activated (26). Our findings show small differences in LVAT between native RBBB and LBBAP (38 vs. 44 ms), which are potentially not even clinically significant considering normal LVAT ranges between 35 and 40 ms (31).

In contrast to the LV activation patterns with LBBAP, data on delayed RV activation during LBBAP are scarce. As LBBAP aims to capture the LBB, the activation waveform needs to propagate from the left to the right ventricle. The exact mechanism of RV activation during LBBAP has not been elucidated, although retrograde invasion of the conduction system is suggested (32). Irrespective of the exact mechanism, RV activation during LBBAP is delayed compared to LV activation (33, 34). This delayed RV activation is characterized by an RBBB pattern on the ECG, which is considered one of the hallmarks of successful LBBAP (17). The delayed RV activation during LBBAP has gained little attention and only a few reports measured RVAT (measured as the interval from pacing stimulus or QRS onset to R wave peak time in lead V1) (26, 28). No previous study assessed the electrocardiographic pattern of delayed RV activation as such. Our results show that with LBBAP, the delayed RV activation encompasses ventricular activation times in between native iRBBB and cRBBB. Despite differences in baseline characteristics in patients with iRBBB, cRBBB and LBBAP, only presence of heart failure was independently associated with longer V1RWPT and V1 R′ duration, but could only partially account for the differences in right ventricular activation times between iRBBB, cRBBB and LBBAP patients.

Of interest, we observed that the delayed RV activation during LBBAP is determined by baseline conduction delay and blocks, but not by the type of LBBAP capture. Indeed, both LBBP and LVSP resulted in similar electrocardiographic characteristics of delayed RV activation. This raises the hypothesis that RV activation with both LBBP and LVSP almost always occurs by activation of the right-sided conduction system capture, as pure myocardial conduction toward the RV would result in ECG characteristics of delayed RV activation resembling more to those seen with cRBBB. Moreover, even in patients with baseline cRBBB, LBBAP further shortens QRS duration, V1RWPT and V1 R′ duration, suggesting that RV activation occurs through the right-sided conduction system. Whether the pacing impulse during LBBAP systematically invades the right-sided conduction system and whether activation of the RV occurs through transseptal activation, or invading connection fibers between the left and right bundle branch or exclusively by retrograde invasion of the left bundle branch needs to be further elucidated (35).

### Long-Term Impact of LBBAP-Induced RV Activation Delay

LBBAP is emerging as a popular pacing modality with growing worldwide adoption. This is mainly explained as LBBAP is characterized by excellent pacing characteristics (low pacing thresholds and high sensing amplitudes), overcoming the main limitations of HBP while still offering a near physiological pacing strategy. The first experience with LBBAP was published in 2017 and several questions remain unanswered regarding the long-term safety, lead performance, feasibility of lead extraction and most important, the long-term clinical outcome. Reports have shown preservation of left ventricular ejection fraction (LVEF) in patients with normal cardiac function undergoing LBBAP and significant improvements in LVEF when LBBAP is implanted in heart failure patients with reduced LVEF (14, 36). However, the follow-up time of these studies was limited, and the impact on right ventricular function have not been considered to date.

During ventricular pacing the normal sequence of electrical activation and electro-mechanical coupling is disrupted. It has been well established that with standard RV apical pacing the delayed activation of the LV can lead to deterioration of the LVEF, pacing-induced cardiomyopathy and adverse outcome including increased mortality (10, 12–14, 17). The pathophysiology of pacing-induced dyssynchrony has been studied during RV apical pacing and can be explained by two observations. First, regions with the earliest activation (i.e., the ventricle which is paced) will contract first, leading to a discoordinated mechanical contraction, reduced ventricular efficiency and increased cavity pressure (37). Secondly, the late-activated segments show increased myocardial work, reduced myocardial blood flow and differences in oxygen consumption and glucose uptake between the first and last activated regions (38). This is a well-known principle in pacing physiology: the ventricle that is first activated exhibits the least myocardial workload, whereas the late-activated ventricle shows increased myocardial workload. Therefore, the delayed RV activation during LBBAP could theoretically result in a higher workload for the RV and might adversely affect the RV over time, but this remains to be explored.

Although both LBBAP and RBBB result in delayed RV activation, global ventricular activation patterns are unlikely to be identical. Indeed, with non-selective LBBP (the most frequently observed LBBAP pacing response during follow-up), direct myocardial capture of the basal septum occurs, which is different from septal activation during RBBB.

To estimate the long-term effects of delayed RV activation by LBBAP, the prognostic outcome of patients with RBBB is sometimes extrapolated to patients with LBBAP. Our results show that the LBBAP-induced delayed RV activation is situated in between incomplete and complete RBBB, and mirrors more closely to iRBBB than to cRBBB. We believe that this observation may be relevant with regard to long-term outcome of LBBAP. First, it shows that with LBBAP the delayed RV activation still occurs by activation of the right-sided conduction system, resulting in only moderate conduction delay and probably a more physiological RV contraction than would be the case with purely myocardial conduction (as the LV experiences during RV apical pacing). Secondly, iRBBB has not been associated with adverse outcome in large population studies. As such, the delayed activation of the RV during LBBAP is unlikely to convey an adverse outcome.

Although it is traditionally accepted that in patients without evidence of cardiac disease, cRBBB is not associated with increased risk of cardiac morbidity or mortality, conflicting data have emerged over the last years about the long-term prognostic significance of incidental cRBBB, especially when cRBBB is associated with heart failure (39). However, very few patients with LBBAP experience cRBBB characteristics with such wide QRS duration, as shown by our results. One group of particular interest in whom LBBAP could result in detrimental effects on the RV are patients with a depressed RV function at the time of LBBAP implant. In these patients, slight delay in RV activation during LBBAP could theoretically result in a further decline of the dysfunctional right ventricle.

The effects of pacing-induced delayed RV activation by LBBAP require careful follow-up and should be addressed in long-term follow-up studies. No assessments of mechanical contraction patterns or function of the RV were performed in this study, although we recognize that long-term follow-up studies with thorough evaluation of the myocardial contraction properties of the RV during LBBAP are needed. Non-invasive ECG imaging or ultra-high frequency ECG might better assess local activation times and depolarization characteristics of specific ventricular segments and could contribute to further insights into the exact mechanism of RV activation during LBBAP.

## Data Availability Statement

The original contributions presented in the study are included in the article/supplementary material, further inquiries can be directed to the corresponding author.

## Ethics Statement

The studies involving human participants were reviewed and approved by University Hospital Ghent. Written informed consent for participation was not required for this study in accordance with the national legislation and the institutional requirements.

## Author Contributions

EO and JD contributed to the conception and design of the article. EO, AD, SC, and JD contributed to the statistical analysis of the article. SK contributed to the literature research. EO wrote the first draft of the manuscript. All authors contributed to manuscript revision, read and approved the submitted version.

## Conflict of Interest

The authors declare that the research was conducted in the absence of any commercial or financial relationships that could be construed as a potential conflict of interest.

## Publisher's Note

All claims expressed in this article are solely those of the authors and do not necessarily represent those of their affiliated organizations, or those of the publisher, the editors and the reviewers. Any product that may be evaluated in this article, or claim that may be made by its manufacturer, is not guaranteed or endorsed by the publisher.

## References

[1] Glikson M Nielsen JC Kronborg MB Michowitz Y Auricchio A Barbash IM. 2021 ESC guidelines on cardiac pacing and cardiac resynchronization therapy. Eur Heart J. (2021) 42:3427–520. 10.1093/eurheartj/ehab364 34586378

[2] Khurshid S Epstein AE Verdino RJ Lin D Goldberg LR Marchlinski FE. Incidence and predictors of right ventricular pacing-induced cardiomyopathy. Heart Rhythm. (2014) 11:1619–25. 10.1016/j.hrthm.2014.05.040 24893122

[3] Khurshid S Liang JJ Owens A Lin D Schaller R Epstein AE. Longer paced QRS duration is associated with increased prevalence of right ventricular pacing-induced cardiomyopathy. J Cardiovasc Electrophysiol. (2016) 27:1174–9. 10.1111/jce.13045 27457998

[4] Kiehl EL Makki T Kumar R Gumber D Kwon DH Rickard JW. Incidence and predictors of right ventricular pacing-induced cardiomyopathy in patients with complete atrioventricular block and preserved left ventricular systolic function. Heart Rhythm. (2016) 13:2272–8. 10.1016/j.hrthm.2016.09.027 27855853

[5] Lamas GA Lee KL Sweeney MO Silverman R Leon A Yee R. Ventricular pacing or dual-chamber pacing for sinus-node dysfunction. N Engl J Med. (2002) 346:1854–62. 10.1056/NEJMoa013040 12063369

[6] Sharma AD Rizo-Patron C Hallstrom AP O'Neill GP Rothbart S Martins JB. Percent right ventricular pacing predicts outcomes in the DAVID trial. Heart Rhythm. (2005) 2:830–4. 10.1016/j.hrthm.2005.05.015 16051118

[7] Sweeney MO Hellkamp AS Ellenbogen KA Greenspon AJ Freedman RA Lee KL. Adverse effect of ventricular pacing on heart failure and atrial fibrillation among patients with normal baseline QRS duration in a clinical trial of pacemaker therapy for sinus node dysfunction. Circulation. (2003) 107:2932–7. 10.1161/01.CIR.0000072769.17295.B1 12782566

[8] Wilkoff BL Cook JR Epstein AE Greene HL Hallstrom AP Hsia H. Dual-chamber pacing or ventricular backup pacing in patients with an implantable defibrillator: the dual chamber and VVI implantable defibrillator (DAVID) Trial. JAMA. (2002) 288:3115–23. 10.1001/jama.288.24.3115 12495391

[9] Teigeler T Kolominsky J Vo C Shepard RK Kalahasty G Kron J. Intermediate-term performance and safety of his-bundle pacing leads: a single-center experience. Heart Rhythm. (2021) 18:743–9. 10.1016/j.hrthm.2020.12.031 33418127

[10] Zanon F Abdelrahman M Marcantoni L Naperkowski A Subzposh FA Pastore G. Long term performance and safety of his bundle pacing: a multicenter experience. J Cardiovasc Electrophysiol. (2019) 30:1594–601. 10.1111/jce.14063 31310410

[11] Huang W Su L Wu S Xu L Xiao F Zhou X. A novel pacing strategy with low and stable output: pacing the left bundle branch immediately beyond the conduction block. Can J Cardiol. (2017) 33:1736e1–3. 10.1016/j.cjca.2017.09.013 29173611

[12] Salden F Luermans J Westra SW Weijs B Engels EB Heckman LIB. Short-term hemodynamic and electrophysiological effects of cardiac resynchronization by left ventricular septal pacing. J Am Coll Cardiol. (2020) 75:347–59. 10.1016/j.jacc.2019.11.040 32000945

[13] Su L Wang S Wu S Xu L Huang Z Chen X. Long-term safety and feasibility of left bundle branch pacing in a large single-center study. Circ Arrhythm Electrophysiol. (2021) 14:e009261. 10.1161/CIRCEP.120.009261 33426907

[14] Vijayaraman P Ponnusamy S Cano Ó Sharma PS Naperkowski A Subsposh FA. Left bundle branch area pacing for cardiac resynchronization therapy: results from the international LBBAP collaborative study group. JACC Clin Electrophysiol. (2021) 7:135–47. 10.1016/j.jacep.2020.08.015 33602393

[15] Vijayaraman P Subzposh FA Naperkowski A Panikkath R John K Mascarenhas V. Prospective evaluation of feasibility, electrophysiologic and echocardiographic characteristics of left bundle branch area pacing. Heart Rhythm. (2019) 16:1774–82. 10.1016/j.hrthm.2019.05.011 31136869

[16] Heckman L Vijayaraman P Luermans J Stipdonk AMW Salden F Maass AH. Novel bradycardia pacing strategies. Heart. (2020) 106:1883–9. 10.1136/heartjnl-2020-316849 33028670

[17] Huang W Chen X Su L Wu S Xia X Vijayaraman P. Beginner's guide to permanent left bundle branch pacing. Heart Rhythm. (2019) 16:1791–6. 10.1016/j.hrthm.2019.06.016 31233818

[18] Alventosa-Zaidin M Guix Font L Benitez Camps M Roca Saumell C Pera G Alzamora Sas MT. Right bundle branch block: prevalence, incidence, and cardiovascular morbidity and mortality in the general population. Eur J Gen Pract. (2019) 25:109–15. 10.1080/13814788.2019.1639667 31339387 PMC6713172

[19] Fahy GJ Pinski SL Miller DP McCabe N Pye C Walsh MJ. Natural history of isolated bundle branch block. Am J Cardiol. (1996) 77:1185–90. 10.1016/S0002-9149(96)00160-9 8651093

[20] Fleg JL Das DN Lakatta EG. Right bundle branch block: long-term prognosis in apparently healthy men. J Am Coll Cardiol. (1983) 1:887–92. 10.1016/S0735-1097(83)80204-6 6826977

[21] Liao YL Emidy LA Dyer A Hewitt JS Shekelle RB Paul O. Characteristics and prognosis of incomplete right bundle branch block: an epidemiologic study. J Am Coll Cardiol. (1986) 7:492–9. 10.1016/S0735-1097(86)80458-2 3950228

[22] Surawicz B Childers R Deal BJ Gettes LS Bailey JJ Gorgels A. AHA/ACCF/HRS recommendations for the standardization and interpretation of the electrocardiogram: part III: intraventricular conduction disturbances: a scientific statement from the American Heart Association Electrocardiography and Arrhythmias Committee, Council on Clinical Cardiology; the American College of Cardiology Foundation; and the Heart Rhythm Society. Endorsed by the International Society for Computerized Electrocardiology. J Am Coll Cardiol. (2009) 53:976–81. 10.1161/CIRCULATIONAHA.108.191095 19281930

[23] Thrainsdottir IS Hardarson T Thorgeirsson G Sigvaldason H Sigfusson N. The epidemiology of right bundle branch block and its association with cardiovascular morbidity—the Reykjavik Study. Eur Heart J. (1993) 14:1590–6. 10.1093/eurheartj/14.12.1590 8131755

[24] De Pooter J Calle S Timmermans F Van Heuverswyn F. Left bundle branch area pacing using stylet-driven pacing leads with a new delivery sheath: a comparison with lumen-less leads. J Cardiovasc Electrophysiol. (2021) 32:439–48. 10.1111/jce.14851 33355969

[25] Chen X Wu S Su L Su Y Huang W. The characteristics of the electrocardiogram and the intracardiac electrogram in left bundle branch pacing. J Cardiovasc Electrophysiol. (2019) 30:1096–101. 10.1111/jce.13956 31094058

[26] Jastrzebski M Kiełbasa G Curila K Moskal P Bednarek A Rajzer M. Physiology-based electrocardiographic criteria for left bundle branch capture. Heart Rhythm. (2021) 18:935–43. 10.1016/j.hrthm.2021.02.021 33677102

[27] De Pooter J El Haddad M Stroobandt R De Buyzere M Timmermans F. Accuracy of computer-calculated and manual QRS duration assessments: clinical implications to select candidates for cardiac resynchronization therapy. Int J Cardiol. (2017) 236:276–82. 10.1016/j.ijcard.2017.01.129 28169058

[28] Jastrzêbski M. ECG and pacing criteria for differentiating conduction system pacing from myocardial pacing. Arrhythm Electrophysiol Rev. (2021) 10:172–80. 10.15420/aer.2021.26 34777822 PMC8576513

[29] Jastrzebski M Burri H Kiełbasa G Curila K Moskal P Bednarek A. The V6-V1 interpeak interval: a novel criterion for the diagnosis of left bundle branch capture. Europace. (2021). 10.1093/europace/euab164 34255038 PMC8742628

[30] Huang T James CA Tichnell C Murray B Xue J Calkins H. Statistical evaluation of reproducibility of automated ECG measurements: an example from arrhythmogenic right ventricular dysplasia/cardiomyopathy clinic. Biomed Signal Process Control. (2014) 13:23–30. 10.1016/j.bspc.2014.03.009 24883077 PMC4036813

[31] Boles U Almuntaser I Brown A Murphy RR Mahmud A Feely J. Ventricular activation time as a marker for diastolic dysfunction in early hypertension. Am J Hypertens. (2010) 23:781–5. 10.1038/ajh.2010.58 20339351

[32] Zhang S Zhou X Gold MR. Left bundle branch pacing: JACC review topic of the week. J Am Coll Cardiol. (2019) 74:3039–49. 10.1016/j.jacc.2019.10.039 31865972

[33] Curila K Jurak P Jastrzebski M Prinzen F Waldauf P Halamek J. Left bundle branch pacing compared to left ventricular septal myocardial pacing increases interventricular dyssynchrony but accelerates left ventricular lateral wall depolarization. Heart Rhythm. (2021) 18:1281–9. 10.1016/j.hrthm.2021.04.025 33930549

[34] Heckman L Luermans J Salden F van Stipdonk AMW Mafi-Rad M Prinzen F. Physiology and practicality of left ventricular septal pacing. Arrhythm Electrophysiol Rev. (2021) 10:165–71. 10.15420/aer.2021.21 34777821 PMC8576493

[35] Lazzara R Yeh BK Samet P. Functional transverse interconnections within the his bundle and the bundle branches. Circ Res. (1973) 32:509–15. 10.1161/01.RES.32.4.509 4702043

[36] Ravi V Hanifin JL Larsen T Huang HD Trohman RG Sharma PS. Pros and cons of left bundle branch pacing: a single-center experience. Circ Arrhythm Electrophysiol. (2020) 13:e008874. 10.1161/CIRCEP.120.008874 33198496

[37] Prinzen FW Hunter WC Wyman BT McVeigh ER. Mapping of regional myocardial strain and work during ventricular pacing: experimental study using magnetic resonance imaging tagging. J Am Coll Cardiol. (1999) 33:1735–42. 10.1016/S0735-1097(99)00068-6 10334450 PMC2041911

[38] Prinzen FW Vernooy K De Boeck BW Delhaas T. Mechano-energetics of the asynchronous and resynchronized heart. Heart Fail Rev. (2011) 16:215–24. 10.1007/s10741-010-9205-3 21103927 PMC3074058

[39] Adesanya CO Yousuf KA Co C Gaur S Ahmed S Pothoulakis A. Is wider worse? QRS duration predicts cardiac mortality in patients with right bundle branch block. Ann Noninvasive Electrocardiol. (2008) 13:165–70. 10.1111/j.1542-474X.2008.00216.x 18426442 PMC6932511

